# A water-soluble polysaccharide from *Grifola frondosa* induced macrophages activation via TLR4-MyD88-IKKβ-NF-κB p65 pathways

**DOI:** 10.18632/oncotarget.21252

**Published:** 2017-09-23

**Authors:** Lihua Hou, Meng Meng, Yuanyuan Chen, Chunling Wang

**Affiliations:** ^1^ Key Laboratory of Food Nutrition and Safety, Ministry of Education, College of Food Engineering and Biotechnology, Tianjin University of Science and Technology, Tianjin 300457, People’s Republic of China

**Keywords:** water-soluble polysaccharide, immunomodulatory, RAW264.7 macrophages, TLR4, NF-κB

## Abstract

Here, the immunomodulatory effects of water-soluble polysaccharide from *Grifola frondosa* on RAW264.7 macrophages and its molecular mechanisms were investigated. *G. frondosa* polysaccharide could obviously enhance immunostimulatory activity such as the release of nitric oxide and cytokine production. Western blotting results showed that *G. frondosa* polysaccharide elevated the TLR4, which might act as an upstream regulator of MyD88 induced *G. frondosa* polysaccharide. MyD88 promoted IKKβ in endochylema and translocate NF-κB p65 subunit into the nucleus which increased the NO production and cytokine/chemokines level. The results suggested that *G. frondosa* polysaccharide activated macrophages through TLR4-MyD88-IKKβ-NF-κBp65 signaling pathways.

## INTRODUCTION

Polysaccharides are a product of living matter, and are widely present in animals, plants and microorganisms. A large number of experiments have shown that polysaccharides have a wide variety of biological functions, such as immune regulation, anti-viral, anti-oxidant, anti-tumor effects, hypoglycemic and lipid-lowering effects [1–5]. Recently, many reports have demonstrated that many polysaccharides are ideal immunomodulators such as *Cordycepstaii* polysaccharides and *Hericium erinaceus* polysaccharide [6]. Polysaccharides could improve the body’s immune system by activating relevant cells and promoting cytokine secretion and antibody production [7]. Moreover, some polysaccharides display multiple pharmacological functions by regulating the immune process [8–10]. Therefore, polysaccharides have broad application potentials in the area of health products because of their good activities, relatively low toxicity and side effects [11].

*Grifola frondosa*, which belongs to the family of *Aphyllophorales* and *Polyporaceae*, has been marketed in China, Japan, and other Asian countries [12]. Fruit bodies and liquid-cultured mycelium from this mushroom have been reported to contain many biologically active compounds [13, 14]. Among all the bioactive components, polysaccharides are the most extensively investigated and have recently attracted considerable attention around the world [15]. Current studies have shown that the polysaccharides from *G. frondosa* (GFPS) possess various effects including antioxidant, antitumor, immunomodulatory, antimicrobial anti-inflammatory, anti-hypertensive, anti-diabetic activities and antiviral activities and so on [16–25]. Our previous work revealed the structural analysis of the polysaccharides from *G. frondosa* and demonstrated it could be activate macrophage RAW 264.7 through increasing the proliferation index and enhancing the immunostimulatory activity such as the cytokine and chemokine production [26].

In this study, in order to elucidate the influence of GFPS induced activation in RAW264.7 cells. The mechanism that GFPS activated macrophages was examined. The production of nitric oxide, the levels of cytokine and chemokine induced by GFPS were measured. The expression of TLR4, MyD88, IKKβ and NF-κB p65 after GFPS treatment were assessed.

## RESULTS AND DISCUSSION

### GFPS induced the morphological changes of RAW264.7 cells by AO staining

**Figure 1:**
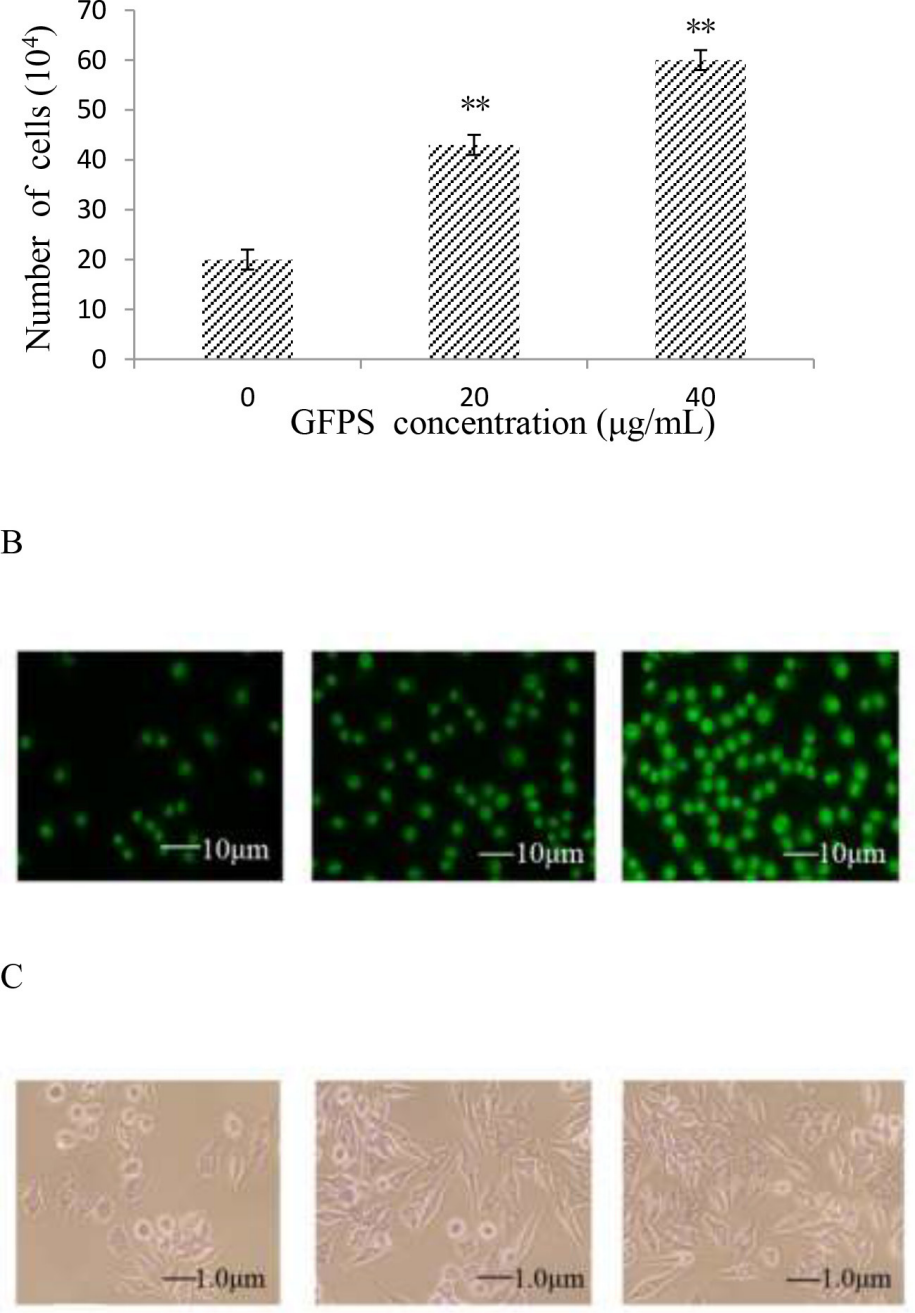

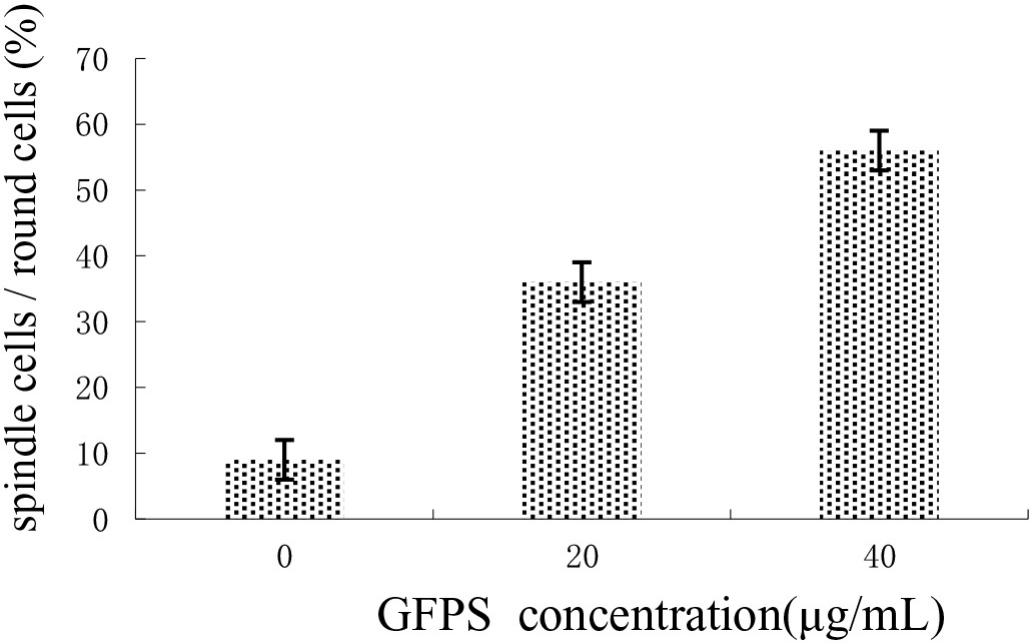
**(A)** The cell number was counted after the treatment with 0, 20 and 40 μg/mL of GFPS; **(B)** AO staining of RAW264. 7 treated with GFPS (×400) (a) Control, (b) 20 μg/mL of GFPS, (c) 40 μg/mL of GFPS; **(C)** PAS staining of RAW264.7 treated with GFPS (×400) (a) Control, (b) 20 μg/mL of GFPS, (c) 40 μg/mL of GFPS; **(D)** the percentage of the round cells and spindle cells after PAS staining of RAW264.7 with 0, 20 and 40 μg/mL of GFPS.

The number of RAW264.7 cells after treatment with 0, 20 and 40 μg/mL of GFPS for 48 h was counted and the data was shown in Figure 1A. The results showed that the number of RAW264.7 cells was enhanced with the increase of the dose of GFPS. Morphological changes of RAW264.7 cells by AO staining were observed and the results were shown in Figure 1B. A typical image of untreated cells with round, intact nuclei was shown in Figure 1Ba. However, cells treated with GFPS increased in cell size visually in Figure 1Bb and 1Bc. The meanintensity of green fluorescence of 0, 20 and 40 μg/mL of GFPS was 11.27, 24.94 and 41.43, respectively. It was also found that at the dose of 40 μg/mL green fluorescence in nuclear area had been enhanced brightness. These results indicated that GFPS could induce RAW264.7 cells activation and affect the level of nucleic acid metabolism.

### GFPS induced the morphological changes of RAW264.7 cells by PAS staining

At the same time, PAS staining of RAW264.7 cells after treatment with different concentrations of GFPS for 48 h was observed. A typical image of untreated cells with round, intact nuclei was shown in Figure 1Ca. However, after treatment with 20 μg/mL and 40 μg/mL GFPS compared with that of the control, the cellular plasma was obviously enhanced (Figure 1Cb and Figure 1Cc) and the number of round cells differentiated into spindle cells was increased. Figure 1D showed that the percentage of the round cells and spindle cells after PAS staining of RAW264.7 with GFPS, suggesting the differentiation of the macrophage cells induced by the GFP. These results indicated that GFPS could stimulate the glycogen metabolism of RAW264.7 cells.

### Effect of GFPS on enzymes activity in RAW264.7 cells

**Figure 2:**
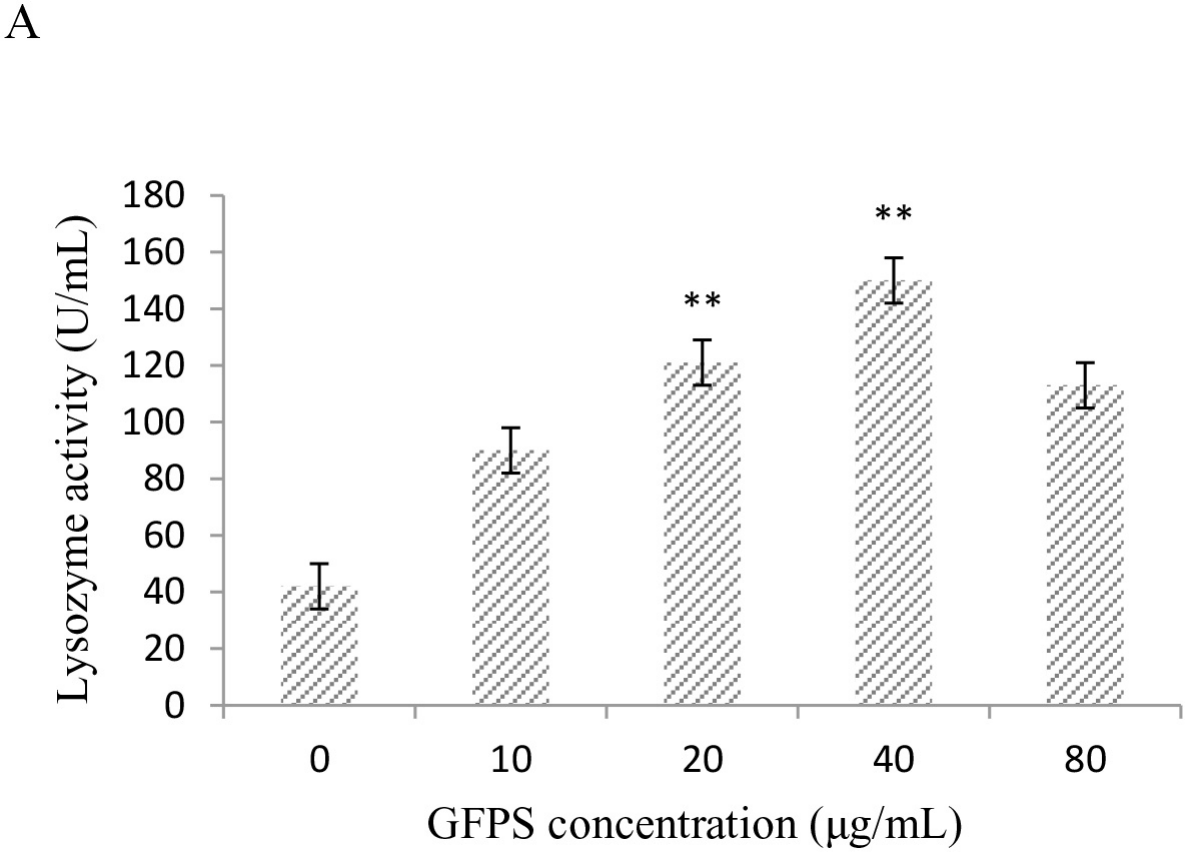

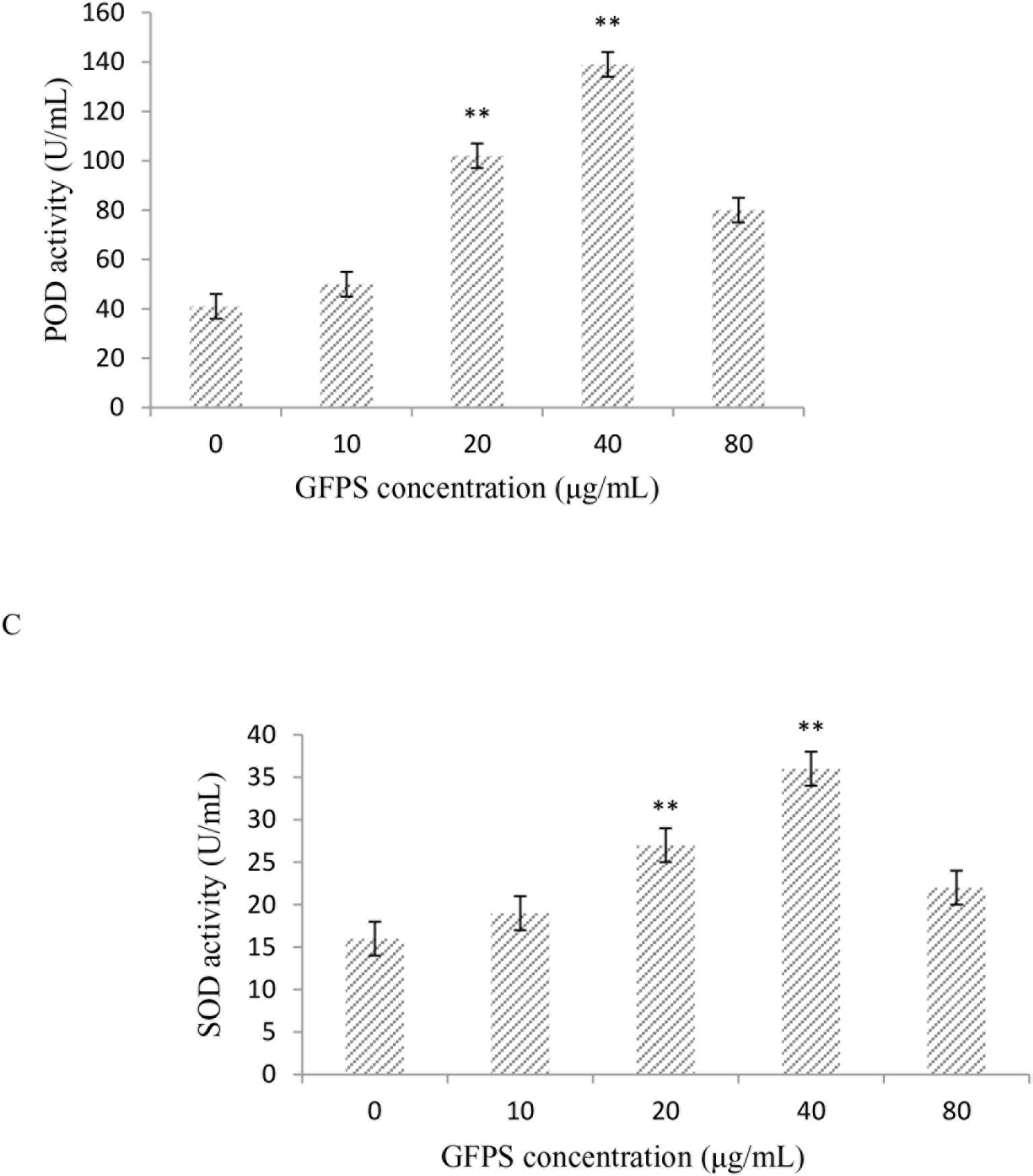
Effects of GFPS on lysozyme activity **(A)**, SOD activity **(B)**, and POD content **(C)**. Values are expressed as mean ± SE. The P values less than 0.05 were considered as significant and indicated by asterisks. Cells were treated with GFPS at the various concentrations (0, 10, 20, 40 and 80μg/mL) for 36 h. (* *p*<0.05, ** *p*<0.01 vs. the control group). The control group was the untreated cells which were incubated with 0μg/mL GFPS. Each bar is representative of three independent experiments, and data were analyzed by ANOVA and Duncan’s multiple range tests.

The lysozyme, superoxide dismutase (SOD) and peroxidase (POD) activity in RAW264.7 cells treated with GFPS at the various concentrations (0, 10, 20, 40 and 80 μg/mL) for 36 h were shown in Figure 2. It was found from Figure 2A that lysozyme activity at 40 μg/mL GFPS was almost four times higher than the control. Figure 2B showed that SOD activity at 40 μg/mL GFPS was more than three times higher than the control. It was also observed from Figure 2A that POD activity at 40 μg/mL GFPS was almost two times more than the control. The results indicated that the lysozyme, SOD and POD activity exhibited a statistically significant increase at 20 μg/mL and 40 μg/mL of this experiment in all GFPS groups compared with the control group. The results suggested that GFP could eliminate excess reactive oxygen species in the body by modulating nonspecific immune enzyme activity to protect the body from environmental damages.

### Effect of GFPS on NO production in RAW264.7 cells

**Figure 3:**
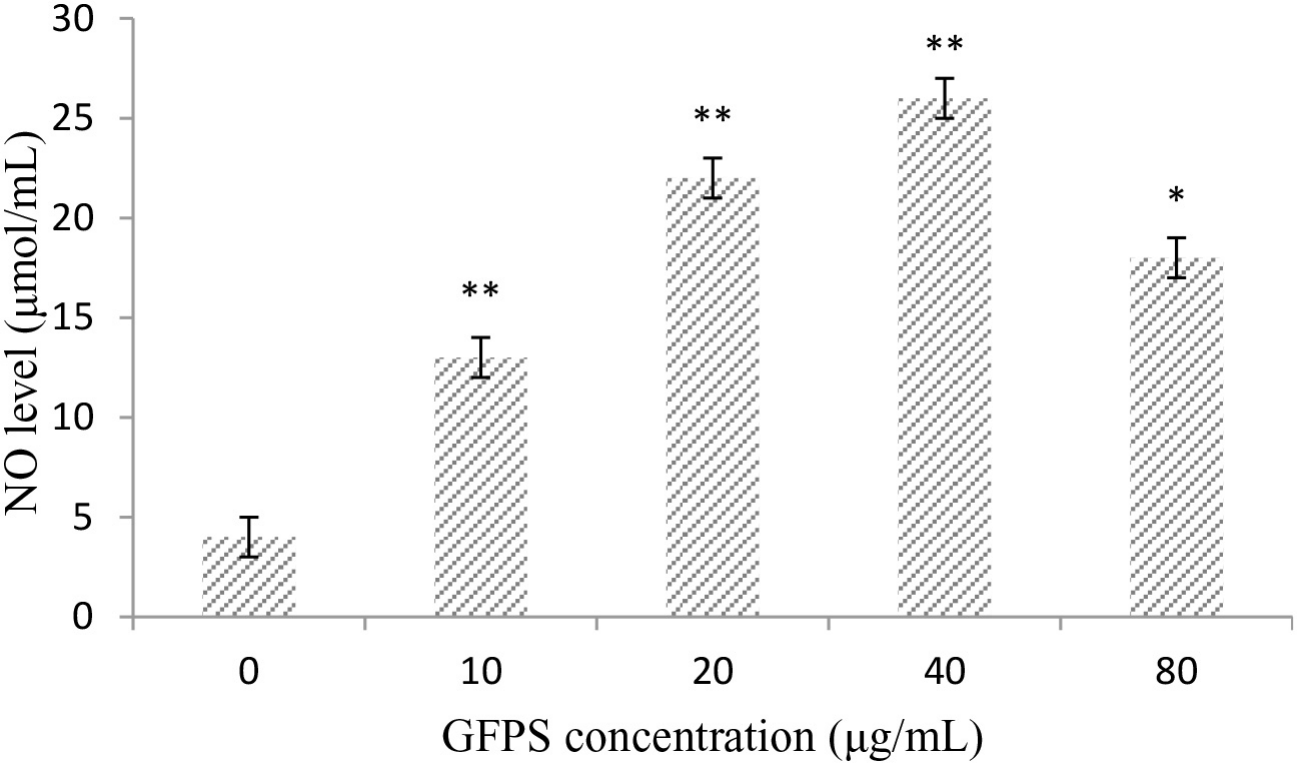
GFPS increased NO production of RAW264.7 cells Cells were treated with GFPS at the various concentrations (0, 10, 20, 40 and 80 μg/mL) for 36 h. (* *p*<0.05, ** *p*<0.01 vs. the control group). The control group was the untreated cells which were incubated without GFPS. Each bar is representative of three independent experiments, and data were analyzed by ANOVA and Duncan’s multiple range tests.

To investigate whether GFPS was capable of inducing NO production from macrophages, RAW264.7 cells were treated with GFPS (0, 10, 20, 40 and 80 μg/mL) for 36 h. As shown in Figure 3, a minimum amount of NO was released in the cells treated with medium alone, whereas cells treated with GFPS at 10, 20 and 40 μg/mL showed significantly (*p*<0.01) higher NO production than that of the control in a dose-dependent manner. Especially, NO productionof RAW264.7 cells treated with GFPS at 40 μg/mL was almost six times more than the control. The results confirmed that the release of NO in RAW264.7 cells could induced by GFPS.

### Effect of GFPS on cytokines and chemokines

**Figure 4:**
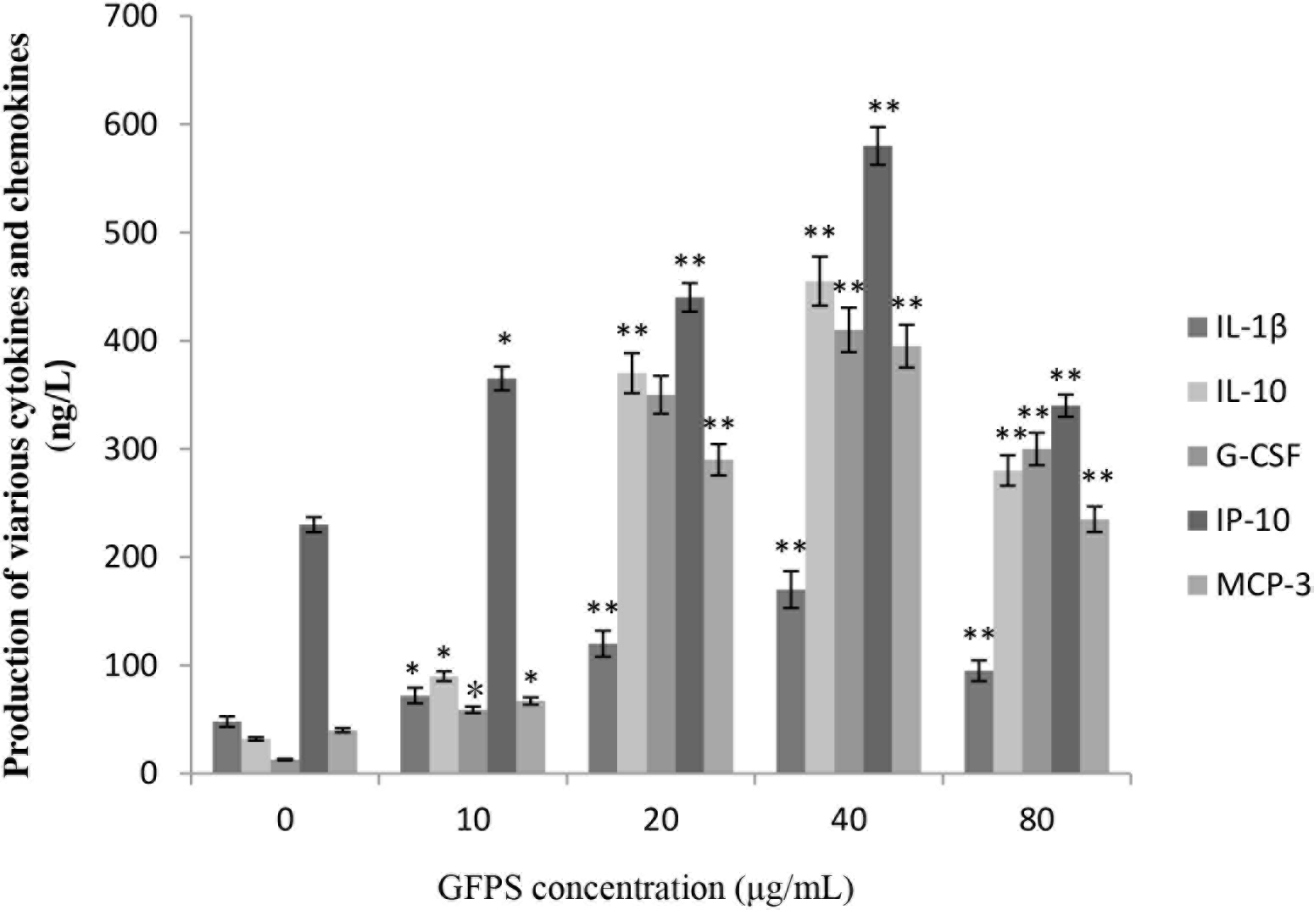
GFPS induced production of various cytokines and chemokines of RAW264.7 cells Cells were treated with GFPS at the various concentrations (0, 10, 20, 40 and 80 μg/mL) for 36 h. (* *p*<0.05, ** *p*<0.01 vs. the control group). The control group was the untreated cells which were incubated with 0 μg/mL GFPS. Each bar is representative of three independent experiments, and data were analyzed by ANOVA and Duncan’s multiple range tests.

Because of the prominent role of cytokine and chemokine in the maturation and function of macrophages, the potential for GFPS to activate the expression of these mediators in RAW264.7 cells were estimated. Figure 4 showed that GFPS induced production of various cytokines and chemokines of RAW264.7 cells treated with GFPS at the various concentrations for 36 h. It was found that the addition of GFPS at 20, 40 and 80 μg/mL resulted in outstanding increased in IL-1β, IL-10, G-CSF, IP-10 and MCP-3 levels in a dose-dependent manner. It was interesting GFPS failed to impact the production of CCL-5 (data not shown). Among the tested samples, that GFPS exhibited the more prominent inhibition profile at the concentration of 40 μg/mL, suggesting that GFPS of 40 μg/mL had the most potent immunomudulatory activity.

### Effect of GFPS on the expression of TLR4, MyD88 and IKKβ

**Figure 5:**
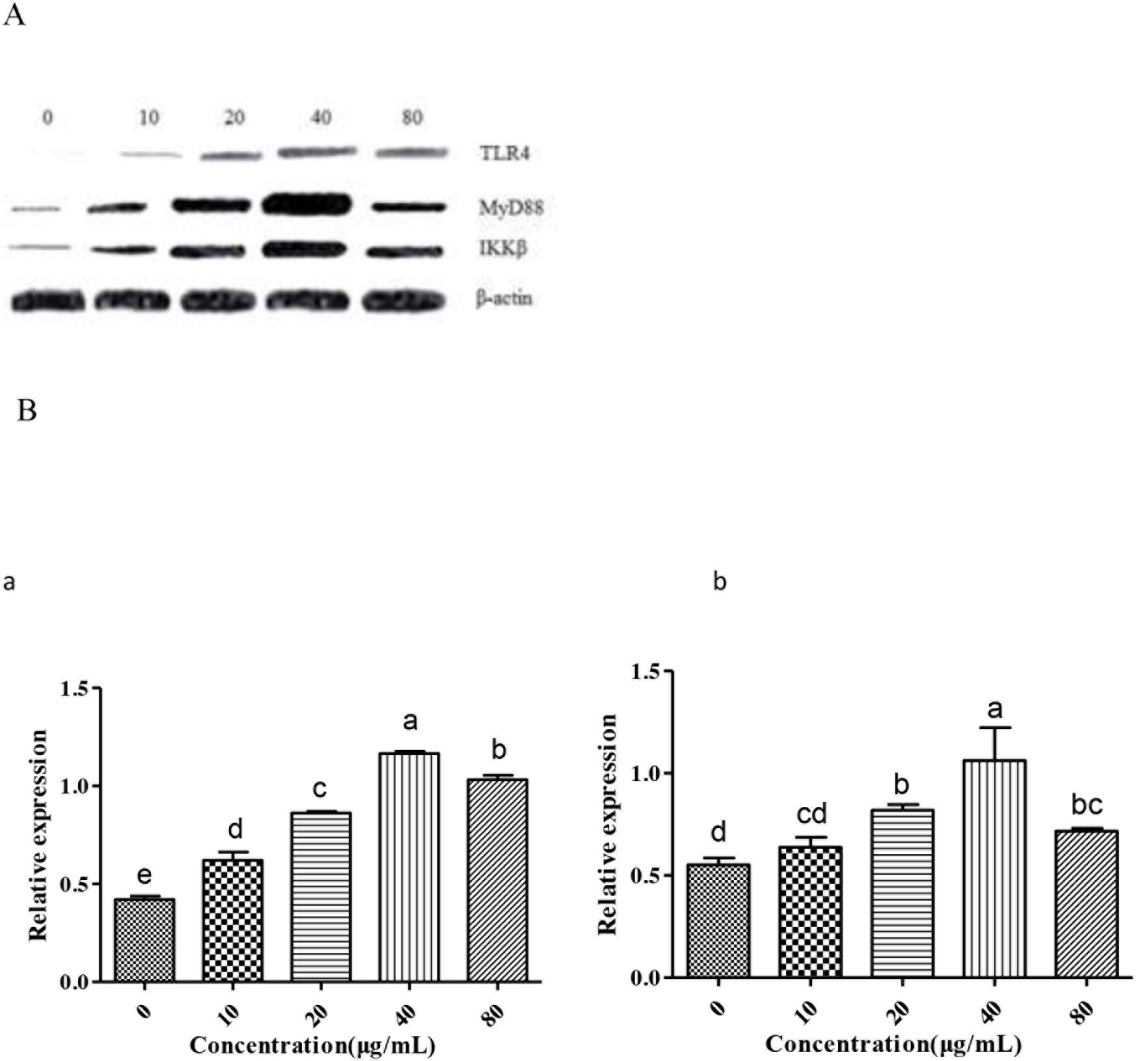

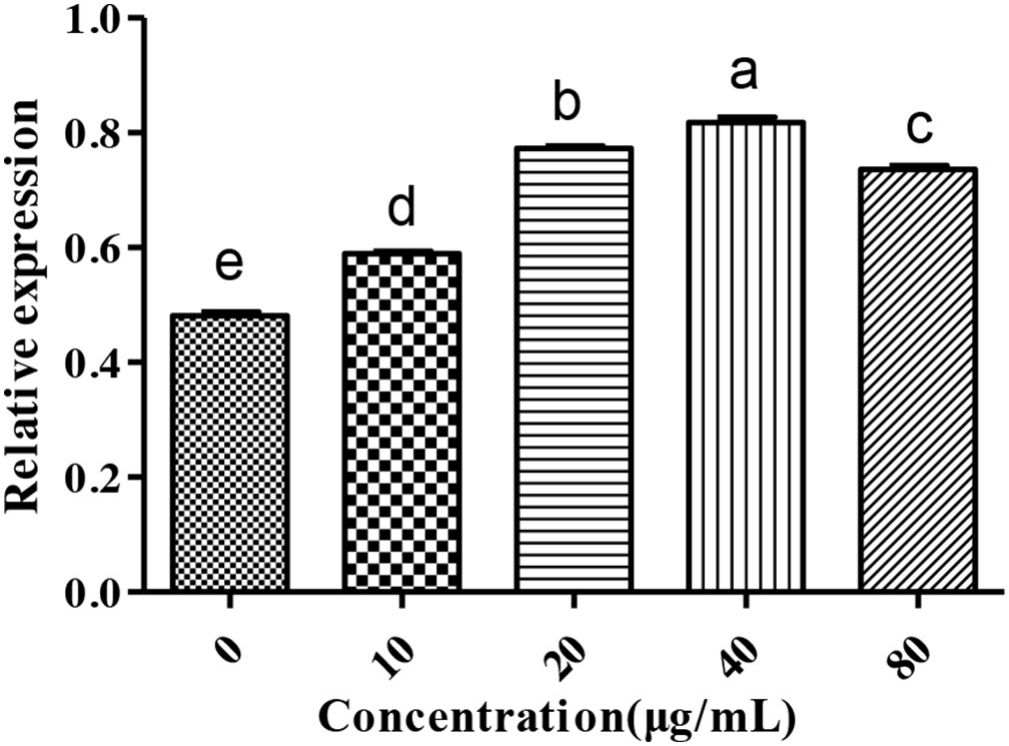
Measurement of TLR4, MyD88 and IKKβ to investigate effect of GFPS on the protein expression of TLR4 and MyD88 in RAW264.7 cells **(A)** Cells were treated with various concentrations (0, 10, 20, 40 and 80 μg/mL) of GFPS for 36 h. **(B)** The analysis of gray intensity of the western blotting (a:TLR4; b: MyD88; c: IKKβ).

We further examined the changes in immunomodulatory responses by analyzing expression of related factors including TLR4, MyD88 and IKKβ. Many studies [27, 28] had shown that TLR4 played an important role in autoimmunological disease. MyD88 is an important intracellular adaptor protein of TLR4. Activation of inhibitor kappa B kinase (IKK) stimulates the translocation and transactivation of transcription factor nuclear factor-kappa B (NF-κB), which induces the expressions of certain inflammatory genes. So the three factors were chosen to be investigated in this study. As shown in Figure 5, the level of TLR4, MyD88 and IKKβ in endochylema increased in a dose-dependent manner, meanwhile, the level of them reached peak value at the concentration of 40 μg/mL.

The results showed that phosphorylated TLR4, MyD88 and IKKβ in endochylema were involved in GFPS-induced RAW264.7 cells activation. In order to further assess the relationship between them, cells were preincubated with TAK-242 (an inhibitor of TLR4) or ST 2825 (an inhibitor of MyD88) prior to adding GFPS (40 μg/mL), and then estimated the expression of phosphorylated TLR4, MyD88 and IKKβ. The level of TLR4 was not reduced by TAK-242 (an inhibitor of TLR4), which indicated that TLR4 was not depended on MyD88. The increase level of p MyD88 was effectively blocked not only by itself inhibitor ST 2825 but also by TAK-242 (an inhibitor of TLR4) in RAW264.7 cells; simultaneously, the expression of IKKβ in endochylema was significantly reversed by ST 2825 (an inhibitor of MyD88). The results indicated that IKKβ expression in endochylema was dependent on MyD88. The results suggested that the effect of GFPS on RAW264.7 cells by combining membrane receptor protein TLR4 induced the upregulation of MyD88, then promoted the expression of IKKβ. GFPS could activate macrophages via TLR4, MyD88 and IKKβ to promote their immunomodulatory activity.

### Effect of GFPS on the expression of NF-κB p65

**Figure 6:**
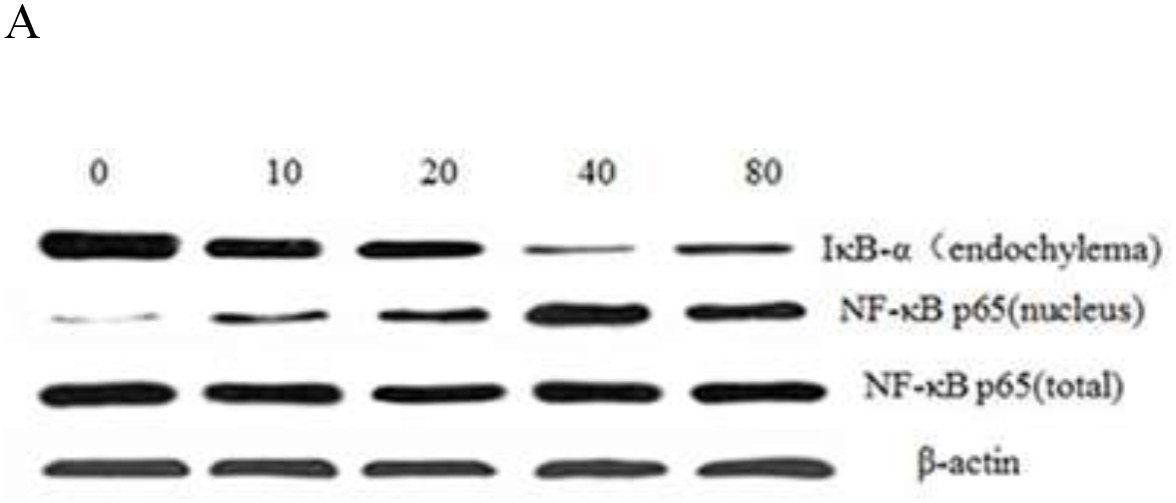

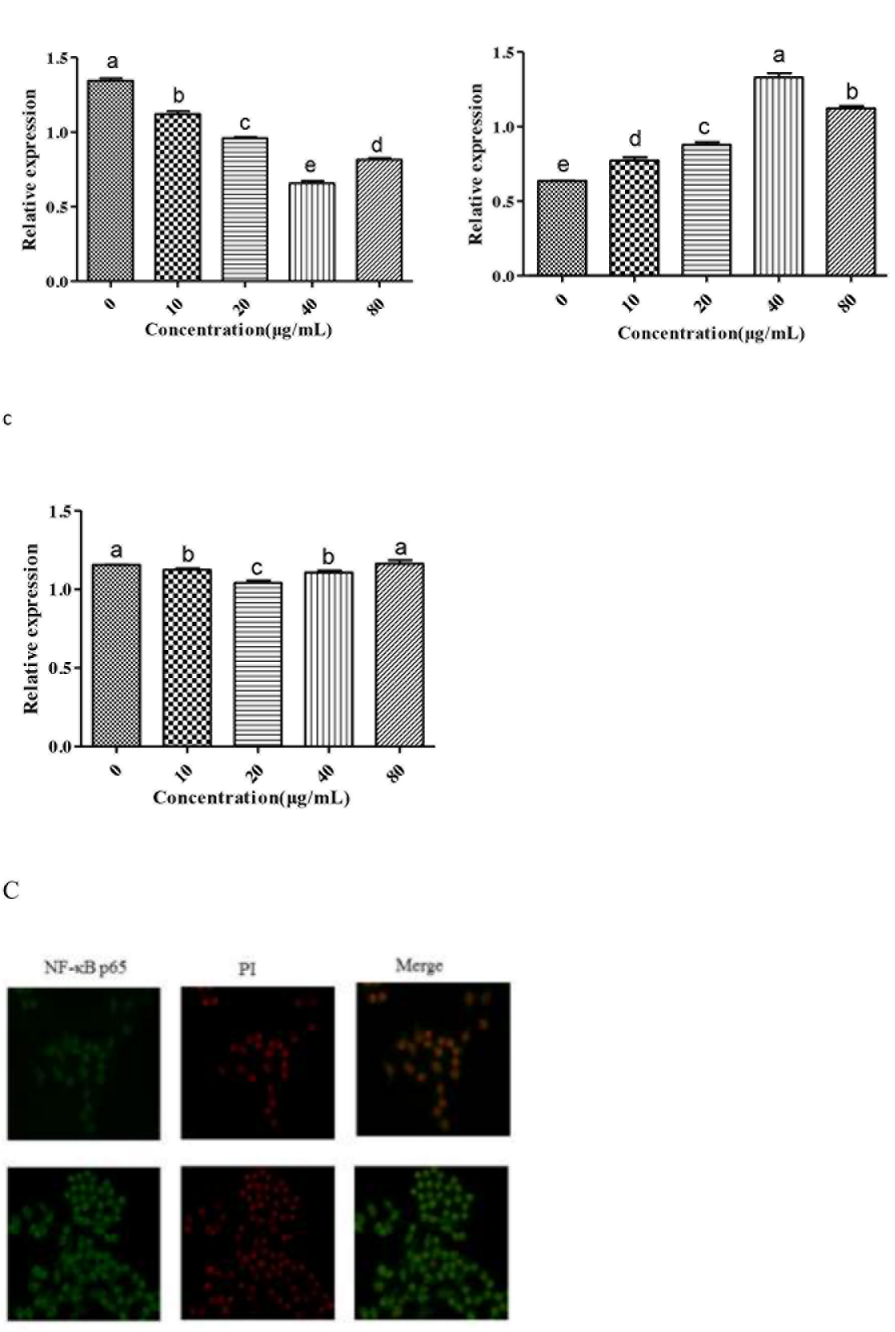
**(A)** Effect of GFPS on the protein expression level of NF-κB p65 and IκB-α in RAW264. 7 cells. Cells were treated with various concentrations (0, 10, 20, 40 and 80 μg/mL) of GFPS for 36 h. The expression of protein was analyzed by western bolt. β-actin was used as an equal loading control. **(B)** The analysis of gray intensity of the western blotting (a: IkB-α; b: NF-kB p65 (nucleus); c: NF-kB p65 (total)). **(C)** Immunofluorescence staining demonstrating the effects of GFPS (40μg/mL) on subcellular localization of NF-κB p65 in RAW264.7 cells. Visualization of the NF-κB p65 fluorescence was recorded by using a LSCM, bar=50μm (×200).

To further investigate whether GFPS activated the NF-κB signaling pathway, the nuclear level of NF-κB p65 subunit and IκB-α was analyzed by the western blotting. Figure 6A was results of the expression of IκB-α and NF-κB p65 and Figure 6B was the analysis of gray intensity of the western blotting. The results indicated that IκB-α proteolytically degraded and free NF-κB p65 translocated into the nucleus. Meanwhile, under the laser scanning confocal microscopy, the intensity of NF-κB p65 fluorescent in the nucleus treated by GFPS was significantly stronger than that of the control group in Figure 6C. It showed that GFPS could induce NF-κB activation in RAW264.7 cells. In short, the all results suggested that NF-κB p65 protein was released into nucleus with IKKβ activation. Transcription of inflammatory gene was induced, which enhanced the binding of nuclear factor p65 and target genes the release of NO. At the same time, the transcription and protein expression level of IL-1β, IL-10 and other related cytokine genes had a certain degree of increase by the activation NF-κB signaling pathway.

## MATERIALS AND METHODS

### Materials

The fruits of *G. frondosa* cultivated in Jiangsu Province, China, were purchased in Qingyuan edible fungi market (Jiangsu, China). The preparation of polysaccharide followed the procedure described in our previous study [26].

### Reagents

Penicillin-streptomycin solution, trypsin, phosphate buffered saline (PBS) and dimethyl sulfoside side (DMSO) were purchased from Thermo (Beijing, China). The multi-plex kit (Cat. MCYTOMAG-70 K-PMX) was obtained from EMD Millipore (Bill- erica, MA, USA). The TLR4, The MyD88, IKKβ, NF-κB p65, β-actin and horseradish peroxidase-conjugated secondary antibodies were purchased from Cell Signaling Technology (Danvers, MA, USA). All other chemicals were of the highest commercial grade available.

### Cell culture

RAW264.7 macrophages were purchased from ATCC (American Type Tissue Culture Collection), and were maintained in RPMI-1640 medium (Solarbio, Beijing, China) supplemented with 10% fetal bovine serum (Gibco BRL, Grand Island, NY, USA),100 units/mL penicillin and 0.1 mg/mL streptomycin at 37°C in a humidified chamber of 95% air and 5% CO_2_ atmosphere.

### Morphological observation by AO staining

RAW264.7 cells were incubated in the absence or presence of GFPS at 37 °C and 5% CO_2_ for 48h. The cells were washed 3 times with PBS and fixed with 4% paraformaldehyde for 10 min at 4°C. Then, cell culture was stained with AO solution (100 μg/mL AO in PBS) at room temperature in the dark. The cells were observed under an inverted fluorescence microscope (AMG, USA).

### Morphological observation by PAS staining

RAW264.7 cells (5 × 10^5^ cells/well) were grown on cover slips in six-well plates and treated with different concentrations of GFPS. Cells were treatment with 10 % (v/v) formalin for 30 min and periodic acid solution for 8 min at room temperature. The cells were washed 3 times with PBS. Then cell culture was stained with Schiff solution for 20min at room temperature in the dark. The cells were observed under an inverted fluorescence microscope (AMG, USA).

### Lysozyme activity assay

Lysozyme activity was determined using commercial kits (Nanjing Jiancheng Bioengineering Institute, Jiangsu, China). Briefly, 200 mL supernatant were added to 1.8 mL of Micrococcus lysodeikticus suspension in phosphate buffer and followed by 15 min reaction at 37°C. And 200 mL reacted suspension was removed into a 96-well plate and measured at 530 nm in a microplate reader (Bio-TEK, USA). Lysozyme activity was defined as mg per ml serum.

### Superoxide dismutase (SOD) activity assay

Serum SOD activity was assayed through commercial test kitspurchased from Nanjing Jiancheng Bioengineering Institute (Nanjing, China). One unit of SOD activity was defined as the amount of enzyme necessary to produce a 50% inhibition of the nitroblue tetrazolium reduction rate measured at 550 nm. SOD activity was expressed as SOD units per ml serum.

### Total peroxidase (POD) activity assay

The total peroxidase activity of serum was measured according to method described in Ref [29]. Briefly, 3, 30, 5, 50-tetramethylbenzidine hydrochloride (TMB, SigmaeAldrich, Germany) and hydrogen peroxide (H_2_O_2_, Sigma-Aldrich) were applied as substrate for peroxidase activity. 15 mL supernatant was diluted to 50 mL in Hanks Balanced Salt Solution (HBSS) without Ca^2+^ and Mg^2+^ in flatbottomed 96 well plates. Then 100 mL of 0.1 mmol/L and 2.5 mmol/L H_2_O_2_ were added and followed by 2 min incubation. Then, 50 mL of 2 mol/L sulphuric acid was added to stop the reaction. The optical density (OD) was read at 450 nm in microplate reader. One unit of peroxidase activity was defined as the amount necessary to produce an absorbance change of 1 OD.

### Measurement of NO production

The nitrite accumulated in culture medium was measured as an indicator of NO production based on the Griess reaction [30]. Briefly, 100 μL aliquots of the supernatant were distributed in a 96-well plate and then equal volumes of the Griess reaction solutions (1% sulfanilamide, 0.1% N-(1-naphthyl)-ethylenediamine dihydro-chloride in 2.5% phosphoric acid) were added. The reaction was allowed to proceed for 15 min at room temperature. The concentration of NO_2_ was calculated by extrapolating a NaNO_2_ standard curve.

### Determination of inflammatory cytokine and chemokine production

Concentrations of interleukin (IL)-1β, interleukin (IL)-10, granulocyte-colony stimulating factor (G-CSF), interferon inducible protein-10 (IP-10), chemokine ligand (CCL)-5 and monocyte chemotactic protein (MCP)-3 were measured by multiplex magnetic bead panel kit (Milliplex murine cytokines/chemokines, Cat. MCYTOMAG-70K-PMX, EMD Millipore, Billerica, MA, USA). Aliquots (25 μL) of cell culture medium were incubated with anti-cytokine or anti-chemokine anti body immobilized beads, detection antibodies, and streptavidin-phycoerythrin according to manufacturer’s instructions. The sample was diluted with medium in order to bring the results into the linear portion of the standard curve. The plate was read by using a MAGPIX® reader running 4.2 xPONENT software (Luminex, Austin, TX, USA). Standards (with a range of 3.2 to 10,000 pg/mL) and high and low concentration quality controls were assayed in duplicate as provided by manufacturer. Data were analyzed using MILLIPLEX™ Analyst software version 3.5.

### Nuclear protein extraction

Nuclear extracts were prepared by lysis nuclei in a high-salt buffer supplemented with protease and phosphatase inhibitors using a nuclear extraction kit (Panomics Inc., Fremont, CA, USA) according to the manufacturer’s protocol. Protein concentrations were quantified using the Bio-Rad protein assay (BCA Protein Assay Kit, Beyotime, Shanghai, China).

### Western blot analysis

After various treatments, RAW264.7 cells were washed three times with cold PBS and lysed with Nuclear and Cytoplasmic Protein Extraction Kit (Beyotime, Shanghai, China). The protein concentrations were quantified using the BCA Protein Assay Kit using bovine serum albumin as a standard. The protocol was described 150 previously and probed with the primary antibodies (Cell Signaling Technology, Danvers, MA, USA). The membranes were washed extensively and incubated with the appropriate secondary antibodies conjugated to horseradish peroxidase (Amersham Pharmacia Biotech). The immunoreactive bands were detected with using an enhanced chemiluminescence (ECL) kit (Millipore Co., Billerica, MA, USA) [31]. The relative protein was β-actin [32]. Each test was performed in triplicate experiments.

### Immunofluorescence imaging for NF-κB p65

Briefly, the cell suspension (1 × 10^5^ cells/well) was inoculated on coverslips that were partitioned previously into a 6-well plate. After 4 h, RAW264.7 cells were treated with 2.4 μM GFPS for 0 or 24 h. Cells were fixed with 3% formaldehyde in PBS for 20 min and washed for three times with PBS. Washed cells were permeabilized using 0.2% Triton X-100 and blocked in 2% bovine serum albumin in PBS. Thereafter, cells were washed for three times with PBS and incubated with the antibody NF-κB p65 (dilution 1: 200) with 2% BSA in PBS at 37°C for 1 h. The resulting cells were washed for three times with PBS and incubated with fluorescein FITC-labeled polyclonal goat anti-mouse IgG antibody (dilution 1: 200) at 37°C for 1 h, after which they were stained with propidium iodide (PI) (Sigma, St. Louis, MO, USA) and scanned by LSCM after being washed with PBS [33]. All images were acquired using the same intensity and photo detector gain.

### Statistical analysis

The data are presented as the means ±S.E. and each experiment was repeated at least three times. Statistical analysis was performed using the SPSS software (version 18.0) to determine the significant differences. All values were analyzed by one-way analysis of variance (ANOVA) followed by a post hoc Tukey’s test for multiple comparisons. Values of *P*<0.05 were considered statistically significant.

## CONCLUSION

In conclusion, this study demonstrated that GFPS had potent immunostimulatory activity in RAW264.7 cells. Results of this study showed that GFPS stimulated lysozyme activity, SOD activity and POD activity, induced NO and inflammatory cytokines/chemokines by macrophages. Meanwhile, GFPS promoted proliferation index of RAW264.7 cells through TLR4, MyD88 and NF-κB signaling pathways. These results further expand current knowledge on mechanism how GFPS acts as a potent adjuvant and agent with immunomodulatory activity.
